# Cultural adaptation, validation, and standardization of a developmental screening tool (ASQ-3) in Iranian children

**DOI:** 10.22037/ijcn.v18i2.39595

**Published:** 2024-03-12

**Authors:** Ghazal SHARIATPANAHI, Roshanak VAMEGHI, Niloufar GHANBARI, Seyed HAMED BAREKATI, Hamid Reza LORNEJAD, Naria ABOLGHASEMI

**Affiliations:** 1 Infectious Department, Bahrami Hospital, School of Medicine, Tehran University of Medical Sciences, Tehran, Iran.; 2 Pediatric Neuro Rehabilitation Research Center, University of Social Welfare & Rehabilitation Sciences, Tehran, Iran.; 3 Pediatrician,Emergency department, Bahrami Hospital, School of Medicine, Tehran University of Medical Sciences, Tehran, Iran.; 4 Pediatrician, Population, Family and School Health Department - Ministry of Health and Medical Education, Tehran, Iran.; 5 Pediatrician, Child Health Office- Ministry of Health and Medical Education, Tehran, Iran.

**Keywords:** Ages and Stages Questionnaire (ASQ-3), Children, Iran, Standardization, Validation

## Abstract

**Objectives:**

This study aimed at culturally adapting, validating, and standardizing the Ages and Stages Questionnaire, third edition (ASQ-3) by implementing a nation-wide cross-sectional methodological study in order to provide a valid and reliable tool for determining the developmental status of Iranian children.

**Materials & Methods:**

This cross-sectional and methodological study was conducted on Iranian children between 1-66 months. The ASQ-3 tool was translated; following that, its face and content validity, as well as the cross-cultural adaptation were assessed by 51 specialists and experts in the field of pediatrics and child development. In order to determine the reliability of the ASQ-3 (using Cronbach’s alpha), and cut-off points. All statistical analyses were performed using STATA software.

**Results:**

This study was enrolled in 2 phases. The face and content validity, as well as the cultural relevance of the Persian version of ASQ-3 was confirmed using panel of specialists views then researchers investigated 11,740 children aged 1-66 months in order to evaluate the reliability of the tool. The Cronbach’s alpha coefficients (reliability) determined for the ASQ-3 and the cut-off points for the ASQ-3 of different age groups and domains were determined by calculating one and two SDs below the mean; the latter represents the main cut-off point, and the interval between the two represents the monitoring zone according to the ASQ-3 technical manual.

**Conclusion:**

The results of this study showed that the Iranian version of ASQ-3 is valid and reliable; moreover, the cut-off points designated for it can be implemented in the Iranian children community to assess their developmental status.

## Introduction

screening is the process by which children are suspected of having developmental delays or disabilities who are in the very early and hidden stages, are detected (1). Primary identification of problems and disabilities is important to increase access to evaluation and intervention (2). Screening child development status has a pivotal role in pediatric care, as primary identification and intervention may affect the path of otherwise persistent problems (3). Therefore, a suitable tool is important to recognize children at risk for developmental delay and those who require a comprehensive assessment. Accordingly, pediatric developmental questionnaires, such as the Ages and Stages Questionnaire (ASQ), meets this requirement (4). 

The ASQ has gained popularity for screening children during examinations due to easy interpretation, short completion time, and easy administration, as well as its usefulness to dramatically improve a clinician’s ability to identify children who are suspected of developmental delays (5). Furthermore, the ASQ is useful for assessing children with biological and environmental risk factors of developmental disorders, such as premature and foster care babies. Additionally, this assessment has a positive influence on promoting parental involvement in their early Child development by itself (6). 

ASQ, the third edition (ASQ-3), is a scale for showing suspected developmental delay of children aged between 1 and 66 months. This tool captures developmental delays in such five domains as fine motor skills, communication, problem-solving, personal-social, and gross motor skills (7). The ASQ-3 has been extensively used in research and clinical settings in many countries, such as the USA (8), Japan (9), and Chile (7), since it is easily administered and has high validity and reliability. It has also been translated into several languages (10) this tool is a parent-rated questionnaire that takes only 10-15 min to complete (11).

In Iran, the second version of ASQ has been translated into Persian, localized, and standardized for more than 15 years ago by the Department of Child Health affiliated to the Ministry of Health, Treatment and Medical Education, University of Social Welfare and Rehabilitation Sciences and the Exceptional Children Education Organization (12). Given the above advantages of the ASQ and the fact that this questionnaire has found its place as a general screening tool in the health system of Iran, those involved in the field of child health and development must use the latest and most complete version of ASQ-3 Accordingly, this study aimed at investigating the cultural adaptation, validation, and standardization of ASQ-3 in Iranian children. 

## Materials & Methods

Study protocol

ASQ-3 was translated into Persian and consisted of 30 items divided into five developmental domains (six items per domain), including communication, gross motor skills, fine motor skills, problem-solving, and personal-social. For each item, the parent was requested to reply “yes” (if their child can do the action), “sometimes” (if their child can sometimes do the action), and “not yet” (if their child cannot do the action). The responses “yes”, “sometimes”, and “not yet” corresponded to the scores of 10, 5, and 0, respectively; therefore, the total score ranges from 0 to 60 for each domain, and a cut-off score was also defined for each domain. 

According to the orders, a score between one and two SDs below the mean is in the “monitoring zone” for which rescreening is suggested. Moreover, a score above two SD below the mean is the referral cut-off point and shows the necessity for additional evaluation. 

The manual for the original ASQ commends that a child is measured as screen positive if his/her score falls below the referral cut-off point in any one of the five domains. The other deficit criterion of failure in at least two domains has also been used in some earlier studies that employ 13-15 items of the current tool with confirmed validity. This software can be used for 10 age groups, including 6-, 12-, 18-, 24-, 30-, 36-, 42-, 48-, 54-, and 60-month cases. 

In order to evaluate the face and content validity, as well as the cultural adaptation of the instrument, 51 experts were selected using the non-random sampling method. Then, using the panel of experts’ opinion method, the viewpoints of them, regarding the face and content validity, as well as the cultural relevance of the the Persian version of ASQ-3 was confirmed, and the second phase of project was enrolled. 

In the second phase, 11,760 Iranian children were selected using the cluster sampling method across the country to determine the reliability (Cronbach’s alpha) and the cut-off points of the ASQ-3. The inclusion criteria were children aged between 1 and 66 months at the time of the study, a minimum level of parents’ education, and giving consent that their child is included in the study. On the other hand, the children with developmental delays in any of the five areas (according to the previous diagnosis of the relevant physicians or therapists) and history of performing any kind of rehabilitation were excluded from the study. 

**Sampling**

Determining the face and content validity, as well as cultural adaptation of the tool: 

The non-random sampling method, which is common in determining the face and content validity of the tools was selected for sampling. Subsequently, the approved and trusted people of the research team, who had the best qualifications and were most familiar with the subject and the tool, were selected to respond. Therefore, 51 experts specialized in pediatrics, child neurology, child development, child psychiatry, child psychology, occupational therapy, and speech therapy, with sufficient teaching experience, research, and work in the health care or rehabilitation system regarding the growth and development of children and children with developmental disorders were selected and included in this study. 

Field execution stage to determine the reliability and cut-off points (Class-cluster):

For sampling, Iran was divided into 7 regions or 7 classes according to a study by Hajian et al. (13) Subsequently, among the provincial centers in each region, one center was randomly selected. Following that, one city was randomly selected out of other cities in each region. Therefore, a total of 14 cities (7 provincial capitals, and 7 other cities) across the country were selected. Afterward, 10 urban and 10 rural clusters were selected out of each city (n=14). In the next step, in each cluster, a household was randomly selected as the herd, and sampling was started from the herd and with comprehensive and detailed instruction that was provided to the questioners, house sampling was continued until reaching designed sample size, out of each cluster and finally each city, the desired number of samples from each age and gender group was (1 girl and 1 boy from each age group in each cluster, a total of 21 girls and 21 boys in each cluster, and a total of 420 girls and 420 boys in each city).

Field execution stage

Considering the stage of determining the face and content validity, as well as the cultural adaptation of the tool, health care experts were invited to participate in a one-day training workshop, and all the steps of the project were taught by the project manager. They were also instructed to transfer the same training to the appropriate, interested, and experienced questioners.

Regarding the field execution stage to determine the reliability and cut-off points, the sampling was conducted in 10 urban and 10 rural clusters in each selected city (n=14) to achieve the required sample size. Upon the first visit to the house of parents with eligible children, they were informed of the research objectives and procedures, written informed consent was obtained from them, and they were provided with the necessary explanations on how to complete the questionnaires. Following that, ASQ-3 questionnaires were randomly distributed among this smaller sample size. Afterward, an appointment was set to collect the completed questionnaires and control the quality of their completion. ASQ-3 translated questionnaires were designed to be printed using computer software, and the text of evolutionary interventions (developmental activities and exercises) recommended by the authors of the ASQ tools were also translated, printed as educational brochures, and distributed among the families during the study. Furthermore, the obtained data were recorded in the portal of the Children’s Health Centers after designing the required software for this purpose. 

ASQ-3 was translated into Persian and provided to the experts for completion. Moreover, this questionnaire was completed by the children’s parents. The completed questionnaires sent to the data collection company were once again checked by the experts of that company in terms of completeness and the absence of obvious contradictions and problems.

Statistical analysis 

The ASQ-3 score range was determined by calculating the mean±SD for each subscale on each ASQ-3 item. For each subscale on each item, and for all statistical analyses, STATA 14.2 (Stata Corp LP, College Station, TX, USA) was used.

Ethical considerations 

The study protocol was reviewed and approved by the Ethics Committees of Tehran University of Medical Sciences, Tehran, Iran. The parents were informed of the research objectives and procedure, and they were also assured that the information obtained would be kept confidential by the implementers of the project. Furthermore, during the research, if the child was diagnosed with developmental delay or disorder, s/he was referred to the relevant medical and rehabilitation centers.

## Results

By applying the viewpoints of experts, the face and content validity, as well as the cultural relevance of the the Persian version of ASQ-3 was confirmed.

The results of the reliability assessment (Cronbach’s alpha coefficient) of the ASQ-3 developmental screening questionnaire by domains and age groups are shown in Table 1.

In communication domain, the lowest reliability values were observed in the 4- (0.377), 6- (0.413), 10- (0.474), and 36-month (0.494) age groups. On the other hand, the highest reliability values were in the 22- (0.724), 24- (0.684), and 2-month (0.67) age groups. Regarding the gross motor skills, the lowest Cronbach’s alpha values were in the 22- (0.345), 24- (0.379), and 18-month (0.383) age groups, and the highest Cronbach’s alpha values were observed in the 14- (0.77), 16- (0.766), 9- (0.740), and 10-month (0.719) age groups. The lowest and highest reliability values based on Cronbach’s alpha in terms of fine motor skills were in the 9- (0.310), 10- (0.360); 2- (0.480), 60- (0.725); as well as 8- (0.724) and 4-month (0.706) age groups, respectively. 

In the same line, considering problem-solving, the lowest reliability values were noted in the 22-(0.384), 18- (0.403), and 20-month (0.420) age groups. In the same domain, the highest reliability values were observed in the 2- (0.752), 8- (0.648), and 4-month (0.646) age groups. Regarding the personal-social domain, the lowest reliability values were in the 18- (0.221), 8- (0. 648), and 30-month (0.416) age groups. Moreover, the highest reliability values in this domain were in the 12- (0.609), 14- (0.592), and 9-month (0.567) age groups.

Table 2 tabulates the cut-off points in the five domains of the ASQ-3. The distance between one SD below the mean (Z-score of -1) and the cut-off point (two SD below the mean) is defined as the monitoring zone. 

developmental delay-need referral, monitoring zone, and normal child were defined according to scores lower than -2 SD from the mean, between 2 and 1 SD, and above -1 SD from the mean, respectively (Table 3 and 4). 

According to the results obtained from the ASQ-3 considering communication, at least and most, 2.3% (18 months) and 7.2% (12 months) of children had lower scores than cut-off points, respectively. In addition, in total age groups, 4.49% of all children had developmental delay in communication, and therefore, needed referral; moreover, 9.17% of cases were in the monitoring zone (Table 3). 

Regarding gross motor skills, at least and most, 9.2% (18-33 months) and 3.8% (9 months) of the children had lower scores than the cut-off points, respectively, which indicates the prevalence of developmental delay in this domain. In addition, in the total age groups, 8.4% of the children had developmental delays, and 97.6% of the cases were in the monitoring zone (Table 3).

Considering the fine motor skills, at least and most, 2.2% (10 months) and 7% (33 months) of children had lower scores than the cut-off points, respectively. In other words, they suffered from developmental delays. In addition, in the total age groups, 4.74% of all children had developmental delay, and therefore, the needed referral. It should be noted that 9.39% of the cases were in the monitoring zone (Table 3).

In the problem-solving domain, the lowest (scores lower than the cut-off point) and highest prevalence rates of developmental delay were 2.3% (10 months) and 7% (14 months), respectively. In addition, in total age groups, 4.4% of all children in the study had developmental delays and needed referral; moreover, 8.38% of the cases were in the monitoring zone (Table 4). Furthermore, the lowest and highest prevalence rates of developmental delay (scores lower than the cut-off point) in the children were 3% (48 months) and 5.7% (4 and 16 months), respectively, in the personal-social domain. In addition, in all age groups, 8.88% of all children had developmental delays and needed referral; additionally, 8.16% of the cases were in the monitoring zone (Table 4).

**Table 1:** Reliability (Cronbach’s alpha coefficient) of the ASQ-3 developmental screening questionnaire

Age group (months)	Developmental domains				
	Communication	Gross motor skills	Fine motor skills	Problem solving	Personal-social
2	0.670	0.620	0.480	0.752	0.531
4	0.377	0.584	0.706	0.646	0.564
6	0.413	0.584	0.644	0.620	0.529
8	0.519	0.625	0.724	0.648	0.450
9	0.591	0.740	0.310	0.483	0.567
10	0.474	0.719	0.360	0.545	0.503
12	0.560	0.709	0.495	0.625	0.609
14	0.568	0.770	0.611	0.466	0.592
16	0.591	0.766	0.614	0.560	0.490
18	0.549	0.383	0.523	0.403	0.221
20	0.660	0.441	0.560	0.420	0.447
22	0.724	0.345	0.539	0.384	0.364
24	0.684	0.379	0.578	0.466	0.426
27	0.645	0.605	0.668	0.517	0.520
30	0.556	0.515	0.678	0.483	0.416
33	0.599	0.502	0.662	0.440	0.504
36	0.494	0.588	0.650	0.485	0.473
42	0.634	0.535	0.559	0.470	0.502
48	0.611	0.591	0.671	0.487	0.466
54	0.530	0.566	0.690	0.465	0.509
60	0.599	0.655	0.725	0.531	0.446

**Table 2:** Cut-off points obtained in the ASQ-3 based on one and two SD below the mean according to the age groups

Age group (months)	Developmental domains															
	Communication			Gross motor skills			Fine motor skills				Problem solving			Personal-social		
	Mean±SD	Z-score of -1	cutting point	Mean±SD	Z-score of -1	cutting point	Mean±SD		Z-score of -1	cutting point	Mean±SD	Z-score of -1	cutting point	Mean±SD	Z-score of -1	Cut-off points
2	50.71±11.36	39.4	28	52.36±10.16	42.2	32	50.94±9.29		41.7	32.4	47.29±13.19	20.9	28	49.31±10.50	38.8	28.3
4	53.20±7.23	46	38.7	52.39±10.73	41.7	30.9	49.72±12.83		36.9	24.1	54.16±9.07	45.4	36	53.64±9.23	44.2	35
6	50.63±8.84	41.8	33	46.38±12.21	34.2	22	54.58±9.38		45.2	35.8	52.92±9.58	43.3	33.8	50.24±10.85	39.4	28.5
8	54.07±8.05	46	38	51.25±11.49	39.8	28.3	57.41±7.75		49.7	41.9	55.40±7.87	47.5	39.7	54.86±7.96	46.9	39
9	52±9.85	42.1	32.3	48.39±13.57	34.8	21.3	56.67±6.78		49.9	43.1	553.93±89.56	45.4	36.8	49.87±11.43	38.4	27
10	53.89±8.41	45.5	37.1	52.23±11.60	40.6	29	57.34±6.28		51.1	44.8	54.96±7.55	47.4	39.9	51.66±9.43	42.2	32.8
12	53.63±8.98	44.7	33.7	50.84±11.67	39.2	27.5	54.75±8.61		46.1	37.5	54.25±8.93	45.3	36.4	51.29±10.86	40.4	29.6
14	52.11±9.98	42.1	32.2	56.29±8.86	47.4	38.6	52.10±9.92		42.2	32.3	55.31±7.08	48.2	41.2	54.75±8.21	46.6	38.4
16	49.71±11.25	38.5	27.2	56.95±8.33	48.6	40.3	53.58±9.52		34.5	27.2	55.43±7.96	47.5	39.5	53.05±8.90	44.2	35.3
18	50.02±10.27	39.8	29.5	58.10±5.53	52.6	47	52.57±9.10		43.5	34.4	51.65±8.61	43	34.4	54.58±6.44	48.1	41.7
20	52.02±11.03	41	30	57.20±7.60	49.6	42	52.90±9.09		43.8	34.7	51.61±8.90	42.7	33.8	53.86±8.12	45.7	37.6
22	51.34±12.17	39.2	27	54.20±7.02	47.2	40.2	48.88±9.84		39	29.2	52.09±8.14	44	35.8	53.81±8.40	45.4	37
24	55.52±8.57	47	38.4	55.64±7	48.6	41.6	50.44±9.75		40.7	30.9	52.26±9.08	43.2	34.1	53.32±8.56	44.8	36.2
27	53.51±10.16	43.4	33.2	52.90±10.03	42.9	32.8	49.29±11.39		37.9	26.5	55.49±7.92	47.6	39.7	50.75±10.14	40.6	30.5
30	54.61±8.33	46.3	38	55.41±8.60	46.8	38.2	48.72±11.57			25.6	55.59±7.58	48	40.4	53.18±8.44	44.7	36.3
33	54.98±9.15	45.8	36.7	55.56±9.21	46.4	37.1	49.71±12.13			25.5	55.58±9.08	46.5	37.4	52.10±10.60	41.5	30.9
36	55.52±7.26	48.3	41	54.97±9.22	45.8	36.5	51.42±11.24			29	55.79±6.91	48.9	42	51.64±9.27	42.4	33.1
42	57±6.56	50.4	43.9	56.15±7.90	48.3	40.4	51.25±10.06			31.1	55.27±7.31	48	40.7	53.30±8.54	44.8	36.2
48	56.23±7.85	48.4	40.5	54.78±8.44	46.3	37.9	48.45±12.46			23.5	53.08±8.72	44.4	35.7	54.34±7.39	46.9	39.6
54	57.91±5.32	52.6	47.3	55.84±7.64	48.2	40.6	50.09±11.66			26.8	47.59±9.76	37.8	28.1	55.84±7.68	48.2	40.5
60	56.22±7.32	48.9	41.6	55.27±8.82	46.5	37.6	46.83±13.16			20.5	47.03±10.57	36.5	26	55.64±7.68	48	40.3

**Table 3:** Frequency and prevalence of developmental delay-need referral, monitoring zone, and normal child based on age groups

Age group (months)	Developmental domains											
	Communication				Gross motor skills				Fine motor skills			
	Need referral N (%)	Monitoring zone N (%)	Normal N (%)	Total N (%)	Need referral N (%)	Monitoring zone N (%)	Normal N (%)	Total N (%)	Need referral N (%)	Monitoring zone N (%)	Normal N (%)	Total N (%)
2	31 (5.6)	43 (7.71)	484 (86.74)	558 (100)	33 (5.9)	60 (10.75)	465 (83.33)	558 (100)	27 (4.8)	83 (14.87)	448 (80.29)	558 (100)
4	14 (2.5)	85 (15.21)	460 (82.29)	559 (100)	36 (6.4)	44 (7.87)	479 (85.69)	559 (100)	31 (5.5)	50 (8.94)	478 (85.51)	559 (100)
6	19 (3.4)	87 (15.56)	453 (86.74)	558 (100)	21 (3.8)	53 (9.48)	485 (86.76)	559 (100)	31 (5.5)	57 (10.20)	471 (84.26)	559 (100)
8	22 (3.9)	70 (12.50)	468 (82.57)	560 (100)	27 (4.8)	31 (5.54)	502 (89.64)	560 (100)	23 (4.1)	8 (1.43)	829 (94.46)	560 (100)
9	26 (4.7)	54 (9.73)	475 (85.59)	555 (100)	46 (8.3)	34 (6.13)	475 (85.59)	555 (100)	16 (2.9)	20 (3.60)	519 (93.51)	555 (100)
10	22 (4)	71 (12.82)	461 (83.21)	554 (100)	33 (6.0)	52 (9.39)	469 (84.66)	554 (100)	12 (2.2)	59 (10.56)	483 (87.18)	558 (100)
12	40 (7.2)	55 (9.84)	486 (86.94)	559 (100)	28 (5.0)	33 (5.90)	498 (89.09)	559 (100)	23 (4.1)	57 (10.20)	479 (85.69)	559 (100)
14	33 (5.9)	52 (9.29)	475 (84.82)	560 (100)	29 (5.2)	29 (5.18)	502 (89.64)	560 (100)	32 (5.7)	65 (11.61)	463 (82.68)	560 (100)
16	27 (4.8)	48 (8.57)	485 (86.61)	560 (100)	34 (6.1)	10 (1.79)	516 (92.14)	560 (100)	27 (4.8)	42 (7.50)	491 (87.68)	560 (100)
18	13 (2.3)	60 (10.73)	486 (86.94)	559 (100)	16 (2.9)	36 (6.44)	507 (90.70)	559 (100)	20 (3.6)	46 (8.23)	493 (88.19)	559 (100)
20	25 (4.5)	70 (12.52)	464 (83.01)	559 (100)	18 (3.2)	10 (1.79)	531 (94.99)	559 (100)	21 (5.6)	43 (7.71)	484 (86.74)	558 (100)
22	38 (6.8)	33 (5.90)	488 (87.30)	559 (100)	25 (4.5)	33 (5.90)	501 (89.62)	559 (100)	20 (3.6)	48 (8.59)	491 (87.84)	560 (100)
24	30 (5.4)	48 (8.57)	482 (86.07)	560 (100)	20 (3.6)	24 (4.29)	516 (92.14)	560 (100)	30 (5.4)	63 (11.25)	467 (83.39)	560 (100)
27	27 (4.8)	31 (5.54)	502 (89.64)	560 (100)	18 (3.2)	78 (13.93)	464 (82.86)	560 (100)	27 (4.8)	55 (9.82)	478 (85.36)	560 (100)
30	23 ( 4.1)	59 (10.54)	478 (85.36)	560 (100)	17 (3.0)	42 (7.50)	501 (89.46)	560 (100)	33 (5.9)	50 (8.93)	477 (85.18)	560 (100)
33	27 (4.8)	52 (9.29)	481 (85.89)	560 (100)	16 (2.9)	46 (8.21)	498 (88.93)	560 (100)	39 (7.0)	39 (6.96)	482 (86.07)	560 (100)
36	35 (6.3)	30 (5.36)	495 (88.39)	560 (100)	32 (5.7)	49 (8.75)	479 (85.54)	560 (100)	24 (4.3)	72 (12.86)	464 (82.86)	560 (100)
42	25 (4.5)	53 (9.46)	482 (86.07)	560 (100)	29 (5.2)	29 (5.18)	502 (89.64)	560 (100)	25 (4.5)	70 (12.50)	465 (83.04)	560 (100)
48	27 (4.8)	20 (7.71)	513 (91.61)	560 (100)	29 (5.2)	52 (9.29)	479 (85.54)	560 (100)	33 (5.9)	68 (12.14)	459 (81.96)	560 (100)
54	22 (3.9)	38 (6.79)	500 (89.29)	560 (100)	39 (7.0)	24 (4.29)	497 (88.75)	560 (100)	35 (6.3)	36 (6.43)	489 (87.32)	560 (100)
60	23 (4.1)	18 (3.22)	518 (92.67)	559 (100)	22 (3.9)	49 (8.77)	488 (87.30)	559 (100)	28 (5.0)	63 (11.27)	468 (83.72)	559 (100)
Total	527 (4.49)	1077 (9.17)	10136 (86.34)	11740 (100)	568 (4.8)	818 (6.97)	10354 (88.19)	11740 (100)	557 (4.74)	1102 (9.39)	10081 (85)	11740 (100)

**Table 4:** Frequency and prevalence of evolutionary delay-need referral, monitoring zone, and normal child based on age groups

Age group (months)	Developmental domains							
	Problem solving				Personal-social			
	Need referral N (%)	Monitoring zone N (%)	Normal N (%)	Total N (%)	Need referral N (%)	Monitoring zone N (%)	Normal N (%)	Total N (%)
2	33 (5.9)	43 (7.71)	482 (86.38)	558 (100)	21 (3.76)	51 (9.14)	486 (87.10)	558 (100)
4	34 (6.1)	60 (10.73)	465 (83.18)	559 (100)	32 (5.7)	28 (5.01)	499 (89.27)	559 (100)
6	22 (3.9)	43 (7.69)	494 (88.37)	559 (100)	21 (3.8)	53 (9.48)	485 (86.76)	559 (100)
8	19 (3.4)	44 (7.86)	467 (88.75)	560 (100)	22 (3.9)	61 (10.89)	477 (85.18)	560 (100)
9	22 (4.0)	80 (14.41)	453 (81.62)	555 (100)	28 (5.0)	39 (7.03)	488 (87.93)	555 (100)
10	13 (2.3)	67 (12.09)	474 (85.56)	554 (100)	24 (4.3)	62 (11.19)	468 (84.48)	554 (100)
12	26 (4.7)	57 (10.20)	476 (85.15)	559 (100)	19 (3.4)	86 (15.38)	454 (81.22)	559 (100)
14	39 (7.0)	22 (3.93)	499 (89.1)	560 (100)	28 (5.0)	59 (10.54)	473 (84.46)	560 (100)
16	21 (3.8)	39 (6.96)	500 (89.29)	560 (100)	32 (5.71)	32 (5.71)	496 (88.57)	560 (100)
18	18 (3.2)	50 (8.94)	491 (87.84)	559 (100)	23 (4.1)	38 (6.80)	498 (89.09)	559 (100)
20	15 (2.7)	64 (11.45)	480 (88.87)	559 (100)	19 (3.4)	75 (13.42)	465 (83.18)	559 (100)
22	28 (5.0)	44 (7.87)	487 (87.12)	559 (100)	21 (3.8)	69 (12.34)	469 (83.90)	559 (100)
24	21 (3.8)	46 (8.21)	493 (88.04)	560 (100)	25 (4.5)	42 (7.50)	493 (88.04)	560 (100)
27	15 (2.7)	45 (8.04)	500 (89.29)	560 (100)	26 (4.6)	60 (10.71)	474 (84.64)	560 (100)
30	30 (5.4)	26 (4.64)	504 (90)	560 (100)	18 (3.2)	31 (5.54)	511 (91.25)	560 (100)
33	18 (3.2)	35 (6.25)	507 (90.54)	560 (100)	28 (5.0)	40 (7.14)	492 (87.86)	560 (100)
36	37 (6.6)	25 (4.46)	498 (88.93)	560 (100)	24 (4.3)	53 (9.46)	483 (86.25)	560 (100)
42	32 (5.7)	39 (6.96)	489 (87.32)	560 (100)	28 (5.0)	26 (4.64)	806 (90.36)	560 (100)
48	36 (6.4)	37 (6.61)	487 (86.96)	560 (100)	17 (3.0)	63 (11.25)	480 (85.71)	560 (100)
54	16 (2.9)	59 (10.54)	485 (86.61)	560 (100)	23 (4.1)	20 (3.57)	517 (92.23)	560 (100)
60	24 (4.3)	59 (10.55)	476 (85.15)	559 (100)	27 (4.8)	21 (3.76)	511 (91.41)	559 (100)
Total	519 (4.4)	984 (8.38)	10237 (87.20)	11740 (100)	1043 (8.88)	958 (8.16)	9739 (82.96)	11740 (100)

## Discussion

A separate and specific approach to developmental assessment in children indicates its special importance (14). In most settings, children’s developmental delays are not diagnosed until school age, and therefore, the untreated remain at the highest risk for academic failure, behavioral problems, as well as emotional and social disorders (15). Since even if young children have an equal opportunity to enter school, those with developmental delays will not have an equal opportunity to take advantage of educational conditions and achieve academic success (16). If there is a delay in several developmental fields in young children, its destructive effects on development will have an increasing effect (17). Therefore, in primary care, the growth delay risk could be ameliorated by the evidence-based benefits of developmental/behavioral promotion. At the same time, the indirect awareness that parents have gained of their child’s abilities and possibly developmental disabilities, compared to other children of the same age by completing the questionnaires, may have resulted in a one-week interval between the test and the retest. Their interaction with their child has improved and this has affected the learning of new skills by the child and of course the test results and has caused more differences and inconsistencies with the test stage.

ASQ-3 can be used to screen and diagnose children as accurately as possible in terms of disorders in motor, communication, and social development (18). The original ASQ-3 was evaluated and established with high specificity, sensitivity, positive predictive value, and negative predictive value in 15,138 North American children (19). Accordingly, it displayed a high specificity and negative prognostic value. In other words, if ASQ-3 had a normal result, it is highly improbable for a child to have a developmental deficit, and it minimized the option of failing to detect children with a definite developmental delay which is one of the main strengths of the questionnaire.

These psychometric possessions were maintained for the native version (20). The reliability values of ASQ-3 on 14,000 American children of various socioeconomic status, as well as different racial groups by the test-retest and the internal consistency methods (Cronbach’s alpha), were estimated at 0.89 and 0.84, respectively. Furthermore, its validity, sensitivity, and specificity were determined at 0.83, 0.81, and 0.83, respectively (21).

The developmental achievement can be different in children based on race, living conditions, and nutrition (22). For example, compared to the Japanese children, US children show that they acquire skills, such as saying a meaningful word, three words, and two-word sentences; taking out clothes; naming four pictures correctly; showing the way at six body parts; walking backward; and making understandable speech more than two months later (9) so cutoff points and normal values of development tools should be different in various communities. Due to the implementation of previous versions of ASQ in development screening in Iran , this study was designed and the results showed that the validity, sensitivity, and reliability of the Persian version of the ASQ-3 was acceptable in most age groups and developmental fields.

The present study is one of the few studies in Iran and even outside Iran to validate and standardize a screening tool for child development in such a population dimension and with accurate sampling representative of the Iranian community in a community-based way at the national level. Moreover, the team of experts who collaborated with this study in the stages of face validity, content validity, and cultural adaptation have been among the best experts in this field in Iran. Furthermore, the multiplicity and variety of specialties used for this stage of work have been observed in fewer similar studies.

The responses given by parents about their children are approximately reliable since inter-observer reliability training reported that the calculation made by health care providers was reliable, particularly if the latter responded to the questions about their children’s present growth (22). Parental explanations considering the differences in socio-economic levels, geographic locations, or parental well-being, provide reliable data about their children’s growth, with test-retest reliability after two weeks above 90% (23). 

Developmental disorder screening is recommended for early intervention in health care settings, and due to the many benefits of the ASQ, it is used as a screening tool in Iran as long as most countries around the world. Therefore, this study evaluated the cultural adaptation, validation, and standardization of the ASQ-3 in Iranian children. The results of the present study showed that the Persian and culturally adapted version of the ASQ-3 had a valid and culturally adopted content; moreover, it had acceptable reliability in most age groups and developmental domains.

## Limitations and Suggestions

In the present study, due to the widespread implementation in the country, direct monitoring of the questioners’ performance was limited, and mistakes or errors were inevitable regarding the questioners’ interaction with families. On the other hand, the reported prevalence of developmental delay in different areas and age groups in the present study was not based on sampling for this purpose (sampling was based on the purpose of tool validation and determination of Iranian cut-off points), and therefore, it may not have provided an accurate estimation of the prevalence of them in the Iranian children’s community, by different local, ethnic, and socio-economic communities. It is suggested that after an appropriate period of time, using standardized questionnaires obtained from this study and Iranian cut-off points with proportional sample size, the developmental status of different age groups of children in different parts of the country can be measured and compared with the results of this study.

## In Conclusion

The ASQ-3 may be recommended for routine pediatric developmental screening, based on given psychometric proper relations and correlations. The results of this study showed that Iranian cut-off points designated for Persian ASQ-3 in the Iranian children community can be implemented and used in this country.

## Authors’ Contribution

Ghazal Shariatpanahi: study conception and design ,supervising data collection, analysis and interpretation of results, and manuscript preparation. 

Roshank Vameghi: study conception and design, suprevising data collection, analysis and interpretation of results. 

Niloufar Ghanbari : manuscript review. 

Seyed Hamed Barekati: supervising data collection, analysis and interpretation of results. 

Hamid Reza Lornejad : supervising data collection, analysis and interpretation of results. 

Naria Abolghasemi : supervising data collection, analysis and interpretation of results.

## Conflict of Interest

No potential conflict of interest was reported by the authors.The research received funding from the Research Vice President of Iran University of Medical Sciences.
